# Azoxystrobin Exposure Impacts on Development Status and Physiological Responses of Worker Bees (*Apis mellifera* L.) from Larval to Pupal Stages

**DOI:** 10.3390/ijms252111806

**Published:** 2024-11-03

**Authors:** Xinle Duan, Huanjing Yao, Wenlong Tong, Manqiong Xiong, Shaokang Huang, Jianghong Li

**Affiliations:** 1 College of Bee Science and Biomedicine, Fujian Agriculture and Forestry University, Fuzhou 350002, China; yhjonline@outlook.com (H.Y.); wenlongtong@sina.cn (W.T.); mqxiong.dingle@outlook.com (M.X.); skhuang@fafu.edu.cn (S.H.); leejh@fafu.edu.cn (J.L.); 2 State Key Laboratory of Ecological Pest Control for Fujian and Taiwan Crops, Fujian Agriculture and Forestry University, Fuzhou 350002, China; 3 Fujian Honeybee Biology Observation Station, Ministry of Agriculture and Rural Affairs, Fuzhou 350002, China

**Keywords:** *Apis mellifera*, azoxystrobin, chronic toxicity, development, gene expression

## Abstract

Honeybee larvae and pupae form the cornerstone of colony survival, development, and reproduction. Azoxystrobin is an effective strobilurin fungicide that is applied during the flowering stage for controlling plant pathogens. The contaminated nectar and pollen resulting from its application are collected by forager bees and impact the health of honeybee larvae and pupae. The current study evaluated the survival, development, and physiological effects of azoxystrobin exposure on the larvae and pupae of *Apis mellifera* worker bees. The field-recommended concentrations of azoxystrobin were found to suppress the survival indices and lifespan in the larval as well as pupal stages; moreover, the rates of the survival and pupation of larvae as well as the body weights of the pupae and newly-emerged adult bees were significantly reduced upon long-term exposure to azoxystrobin. In addition, azoxystrobin ingestion induced changes in the expression of genes critical for the development, immunity, and nutrient metabolism of larvae and pupae, although the expression profile of these genes differed between the larval and pupal stages. Results indicated the chronic toxicity of azoxystrobin on the growth and development of honeybee larvae and pupae, which would affect their sensitivity to pathogens and other external stresses during the development stage and the study will provide vital information regarding the pollination safety and rational use of pesticides.

## 1. Introduction

Honeybees are indispensable pollinators of wild plants and crops, promoting plant reproduction as well as maintaining biodiversity and ecological balance, thereby ensuring human welfare [1,2]. The pollination services provided globally by pollinators in agriculture are currently valued at over 400 billion USD per year [3]. However, the survival and pollination services of honeybees are threatened by various factors, particularly the toxicity of pesticides, which are chiefly responsible and a key driver for the decline of honeybees at the individual as well as colony levels [2,3]. The continued decline of pollinators is expected to threaten pollination services that are crucial for crop production and the maintenance of natural ecosystems, and even food security; thus, the risks associated with the exposure of honeybees to pesticides have attracted growing attention and concern [1,2,3].

Fungicides represent a vital category of plant protection products that are widely applied for the prevention and control of fungal plant diseases and mycotoxin contamination of food and feed products; they contribute to at least 35% of the pesticide market globally and their share is predicted to increase in the future [4]. Acute toxic effects of the vast majority of fungicides on honeybees, as evaluated via lab tests, and obvious adverse effects on flower-visiting behaviors have hitherto not been observed [5]. Thus, honeybees and other wild bees are inevitably exposed to various fungicides, applied for the control of plant disease and pests, in a variety of ways during pollination and foraging activities [3,5]. In fact, an increasing number of studies have confirmed that not all fungicides are safe for pollinators [2,3,5]. The continuous application of pesticides is likely to lead to the accumulation of their active ingredients and residues in various organs and secretions of plants, including pollen and nectar, which are important food resources for the honeybees [2,3,4]. These contaminated food resources are transported back to the colony by the foragers, resulting in the spread of the contamination throughout the bee colony; Moreover, the consumption of the contaminated food resources by adults and larvae are considered the most important exposure route for both honeybees and non-*Apis* bees [6]. The consumption of an artificial diet containing chlorothalonil (30 or 100 mg/L) resulted in chronic toxicity and thereby, significantly lower survival rates of the honeybee larvae [7]. Difenoconazole significantly reduced the survival rate of adult stingless bees (*Melipona scutellaris*), resulting in mortality within 72 h [8], while this fungicide inhibited mitochondrial respiration and caused significant damage to the mitochondrial DNA (mtDNA) in bumblebees (*Bombus terrestris* L.) both in vitro and in vivo conditions [9]. The fungicide Pristine^®^ not only reduced the lifespan of worker bees and the population size of the colony, but also led to protein malnutrition in individual bees, affecting their forage preferences and behavior [5]. The exposure of adult bees to the fungicide carbendazim altered the expression pattern of 266 proteins, 218 of which were down-regulated; these proteins are closely associated with the development, nutrition, immunity, and behavior of honeybees [10].

Azoxystrobin is a member of the strobilurin group fungicides and has been widely used in agriculture since 1996 for the control of various diseases that impact pollen and nectar plants such as rape, alfalfa, sunflower, and lychee [11]. The action mechanism of azoxystrobin involves the inhibition of mitochondrial respiration by hindering electron transport at the Q_0_ site of the cytochrome b subunit of mitochondrial complex III, thus affecting the synthesis of ATP [2,11,12]. The high efficiency, low toxicity, broad-spectrum activity, and improved safety profile of azoxystrobin compared to previously used fungicides have contributed to the steady increase in the sales of azoxystrobin year on year [1,13]. Seven commercial formulations of azoxystrobin are currently registered for plant disease control in China, with azoxystrobin suspension concentrate (SC, 250 g/L) employed as the main commercial product as per the pesticide registration database of China [14]. Although its target is plant pathogenic fungi, azoxystrobin can also affect other eukaryotes [13,15,16]. On exposure to environmental levels of azoxystrobin, the anxiety-like behavior of male zebrafish was increased and the levels of reactive oxygen species (ROS), and malondialdehyde (MDA), as well as the activities of catalase (CAT), superoxide dismutase (SOD), and acetylcholinesterase (AChE) were significantly altered [15]. In addition, azoxystrobin induced oxidative stress leading to damage in earthworm (*Eisenia fetida*), with significant correlation observed between exposure concentration and ROS content [16]. The United States Environmental Protection Agency reported low acute toxicity of azoxystrobin for adult bees (*Apis mellifera*) in 1997 [17]. However, azoxystrobin exerted sublethal effects in the midgut of adult workers (*A. mellifera*) causing cytoplasm vacuolization and increased cellular fragmentation in the gut [12]. Additionally, azoxystrobin residues have been reported in bee foods, such as nectar, pollen, royal jelly, and bread, as also in the tissues of honeybee from various countries, including the United States, Germany, and others [18]; these residue negatively impact the development, physiology, and behavior of adult worker bees [1,12].

Honeybees, like most typical insects, undergo complete metamorphosis during their life cycle that involves four developmental stages: egg, larva, pupa, and adult [3]. The ecdysone receptor (*ecr*) and ultraspiracle protein (*usp*) play key roles in ecdysone signal transduction in insects during metamorphosis that in turn activates downstream transcription factors and regulates molting-associated physiological responses and changes in metabolism [19]. Hexamerin is a key functional storage protein in insects that is used for the reconstruction of the tissues and structures in the pupa; it also directly affects the growth, development, nutritional status, and immunity of larvae and pupae [20]. Hexamerin 70b (*hex70b*) compensates for the insufficient dietary intake of proteins in the pupal stage and responds to the altered levels of juvenile hormone, while hexamerin 110 (*hex110*) is recycled for the amino acid demand of adult tissue formation as metamorphosis proceeds [20,21]. The insulin/insulin-like signaling pathway (IIS) reflects the nutritional and development status of the worker bee at various stages of development, and the insulin-like peptide 1 (*ilp1*) and 2 (*ilp2*) play roles in the lipid metabolism and nurse-to-forager behavior transition in adult bees [22]. Vitellogenin (*vg*) is another important protein that is associated with longevity, reproduction, antiaging, and behavioral maturation of honeybees; it functions as an antioxidant that synergizes with the protective enzymes SOD and CAT in response to oxidative stress induced by pyrazoxystrobin and other fungicides [2,3,23]. The antimicrobial peptides (AMPs) *apidaecin*, *abaecin*, *defensin1,* and *hymenoptaecin* are key components of the innate immunity of honeybees against microbial pathogens and environmental stress; however, their expression could be affected by pesticides and other external factors, potentially increasing honey bees susceptibility to pathogens [2,3]. To date, several investigations have ascertained the negative effects of azoxystrobin on adult worker bees and the accumulation of high levels of the fungicide residue in beehives. However, the chronic toxicity of azoxystrobin on the development and physiology of honeybee larvae and pupae has been rarely reported. Both the larvae and pupae live only in the comb, and nearly all bee larvae require a diet relatively rich in royal jelly or pollen, consuming substantially greater quantities than the adults [6]. Thus, a contamination of the honeycomb, royal jelly, or pollen with fungicide residue would certainly pose a threat to the health of the larvae and pupae.

The aim of the current study was to evaluate the potential adverse effects of azoxystrobin on the development as well as on the nutritional and immune status of honeybee larvae and pupae. Healthy 1-day-old larvae were exposed to the field-recommended concentrations of azoxystrobin until eclosion, and the survival rate, body weight, and other developmental parameters of worker bees were recorded from larval to adult stage with the dead individuals being removed daily. The level of ROS and the activities of the enzymes SOD and CAT in the larvae and pupae were also detected. Moreover, the relative expression level of genes associated with development (*ecr* and *usp*), nutrition (*ilp1*, *ilp2*, *hex70b*, *hex110,* and *vg*), and immunity (*abaecin*, *apidaecin*, *defensin1,* and *hymenoptaecin*) in both larvae and pupae were detected. The current study provides novel evidence for the potential adverse effect of azoxystrobin exposure on the health of honeybees and a theoretical basis for achieving the balance between pollination safety and pesticide use that is expected to contribute to the comprehensive assessment of the ecotoxicology and environmental safety of azoxystrobin.

## 2. Results

### 2.1. Effect of Azoxystrobin Exposure on the Rates of Survival, Pupation, and Emergence of Larvae and Pupae

In all four azoxystrobin exposure treatments, the survival rate and pupation rate of larvae decreased with increasing exposure concentrations (one-way ANOVA, for survival rate: F(4,15) = 100.2, *p* < 0.0001; for pupation rate: F(4,15) = 45.99, *p* < 0.0001, Table 1). Further post hoc analyses showed that the survival (87.36%, 82.31%, and 67.83%) and pupation (67.55%, 56.72%, and 48.15%, respectively) rates of larvae in the 167, 250, and 313 mg/L treatment groups, respectively, were significantly lower than the control group (survival rate: for 167 mg/L group, *p* = 0.0010; for 250 mg/L group, *p* < 0.0001; for 313 mg/L group, *p* < 0.0001; pupation rate: for 167 mg/L group, *p* = 0.0426; for 250 mg/L group, *p* < 0.0001; for 313 mg/L group, *p* < 0.0001). However, the emergence rate did not vary significantly between the treatment and control groups (F (4,15) = 1.19, *p* = 0.3535). Azoxystrobin exerts considerable effects associated with chronic toxicity in *A. mellifera*, suppressing the survival indicators and lifespan of both larvae and pupae. 

### 2.2. Effect of Azoxystrobin Exposure on the Body Weight of Pupae and Newly-Emerged Adult Bees

The toxicity effects of azoxystrobin on the body weight of both pupae and the newly-emerged adult bees were ascertained subsequently, and revealed significant weight reduction in both upon exposure to azoxystrobin (one-way ANOVA, for pupa, F(4,15) = 59.45, *p* < 0.0001; for newly-emerged adult bee, F(4,15) = 73.23, *p* < 0.0001). Compared with the control group, the weight of pupae reduced by 7.65%, 8.17%, 17.77%, 26.74% in each azoxystrobin treatment group, respectively (for 125 mg/L group, *p* = 0.0077; for 167 mg/L group, *p* = 0.0045; for 250 mg/L group, *p* < 0.0001; for 313 mg/L group, *p* < 0.0001). Similarly, the weight of newly-emerged adult bees also reduced by 13.95%, 22.60%, 29.81%, 35.53%, respectively (for 125 mg/L group, *p* = 0.0007; for 167 mg/L group, *p* < 0.0001; for 250 mg/L group, *p* < 0.0001; for 313 mg/L group, *p* < 0.0001). Furthermore, the reduction in body weight exhibited a correlation with the concentration of azoxystrobin.

### 2.3. Expression Level of Development-Related Genes in Both Larvae and Pupae of A. mellifera Worker

**Figure 1:**
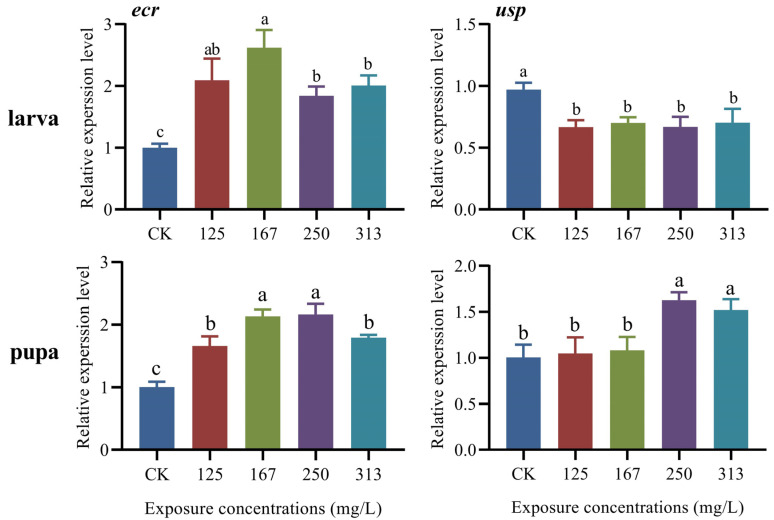
Variable relative expression level of two development-related genes (*ecr*, *usp*) in both larvae and pupae of *Apis mellifera*. Azoxystrobin interfered with the expression of *ecr* and *usp* in both larvae (one-way ANOVA, *ecr*, F(4,10) = 20.24, *p* < 0.0001; *usp*, F(4,10) = 8.94, *P* = 0.0024) and pupae (one-way ANOVA, *ecr*, F(4,10) = 44.91, *p* < 0.0001; *usp*, F(4,10) = 14.08, *p* = 0.0004).The data in the figure are mean ± SE (standard error) and the different lowercase letters above bars indicate significant difference among different azoxystrobin exposure treatments (*p* < 0.05).

Exposure to azoxystrobin significantly induced the expression of *ecr* in larvae (one-way ANOVA, F(4,10) = 20.24, *p* < 0.0001, Figure 1); in particular, the treatment group exposed to 167 mg/L azoxystrobin exhibited the highest expression which was 2.61-fold higher than that of the control group (for 125 mg/L group, *p* = 0.0011; for 167 mg/L group, *p* < 0.0001; for 250 mg/L group, *p* = 0.0072; for 313 mg/L group, *p* = 0.0020). Furthermore, the expression of *usp* was significantly lower in the larvae exposed to azoxystrobin compared to the control group (F(4,10) = 8.94, *p* = 0.0024), indicating that azoxystrobin had a significant inhibitory effect on the expression of the *usp* gene in larvae. The expression levels of *ecr* (F(4,10) = 44.91, *p* < 0.0001) and *usp* (F(4,10) = 14.08, *p* = 0.0004) in the pupae exposed to azoxystrobin were significantly higher than those in the control group. Among the different treatment groups, the expression of *ecr* and *usp* significantly increased by 2.16-fold and 1.62-fold, respectively, in the group exposed to 250 mg/L of azoxystrobin (*ecr*: for 125 mg/L group, *p* = 0.0004; for 167 mg/L group, *p <* 0.0001; for 250 mg/L group, *p* < 0.0001; for 313 mg/L group, *p* < 0.0001; *usp*, for 125 mg/L group, *p* = 0.9957; for 167 mg/L group, *p* = 0.9554; for 250 mg/L group, *p* = 0.0016; for 313 mg/L group, *p* = 0.9963).

### 2.4. Expression Level of Immune-Related Genes in Both Larvae and Pupae of A. mellifera Worker

**Figure 2:**
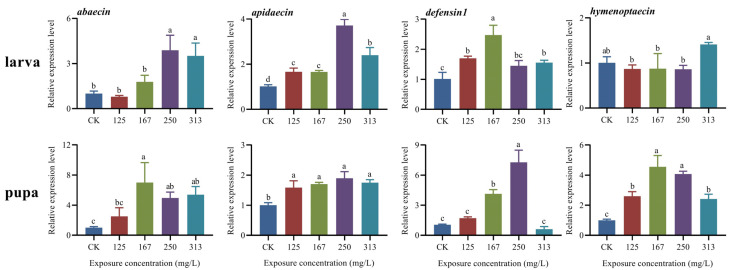
Variable relative expression level of four immune-related genes (*abaecin*, *apidaecin*, *defensin1*, *hymenoptaecin*) in both larvae and pupae of *Apis mellifera*. Azoxystrobin disturbed the expression of *abaecin*, *apidaecin*, *defensin1,* and *hymenoptaecin* in both larvae (one-way ANOVA, *abaecin*, F(4,10) = 15.58, *p* = 0.0003; *apidaecin*, F(4,10) = 71.56, *p* < 0.0001; defensin1, F(4,10) = 21.59, *p* < 0.0001; and hymenoptaecin, F(4,10) = 5.66, *p* = 0.0121) and pupae (one-way ANOVA, *abaecin*, F(4,8) = 9.60, *p* = 0.0038; *apidaecin*, F(4,10) = 15.29, *p* = 0.0003; *defensin1*, F(4,10) = 65.54, *p* < 0.0001; and *hymenoptaecin*, F(4,10) = 38.47, *p* < 0.0001).The data in the figure are mean ± SE (standard error) and the different lowercase letters above bars indicate significant difference among different azoxystrobin exposure treatments (*p* < 0.05).

Exposure to azoxystrobin caused significant variation in the expression of four genes associated with the immune response in the larvae and pupae of honeybees (Figure 2). Azoxystrobin treatment up-regulated the expression of *abaecin* (one-way ANOVA, F(4,10) = 15.58, *p* = 0.0003), *apidaecin* (F(4,10) = 71.56, *p* < 0.0001), and *defensin1* (F(4,10) = 21.59, *p* < 0.0001) in the larvae. Similarly, azoxystrobin disturbed the expression of *hymenoptaecin* in larvae (F(4,10) = 5.66, *p* = 0.0121), which was up-regulated only in the 313 mg/L treatment group, although the difference with the control group was not statistically significant (for 125 mg/L group, *p* = 0.8549; for 167 mg/L group, *p* = 0.8796; for 250 mg/L group, *p* = 0.8369; for 313 mg/L group, *p* = 0.0912). In addition, up-regulated expression of *abaecin* (F(4,10) = 9.60, *p* = 0.0038), *apidaecin* (F(4,10) = 15.29, *p* = 0.0003), and *hymenoptaecin* (F(4,10) = 38.47, *p* < 0.0001) was observed in the pupae of the treatment groups. However, the expression of *defensin1* was only significantly up-regulated in the 167 mg/L and 250 mg/L fungicide treatment groups (for 125 mg/L group, *p* = 0.6574; for 167 mg/L group, *p* = 0.0006; for 250 mg/L group, *p* < 0.0001; for 313 mg/L group, *p* < 0.0001). 

### 2.5. Expression Level of Nutrient-Related Genes in Both Larvae and Pupae of A. mellifera Worker

**Figure 3:**
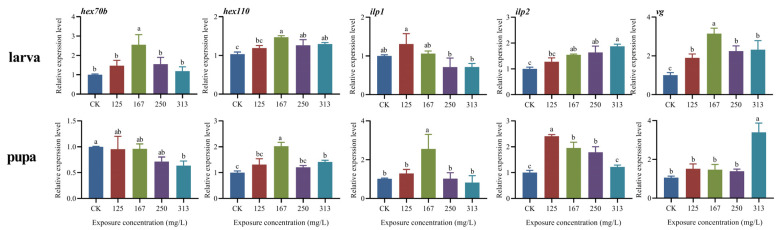
Variable relative expression level of five nutrient-related genes (*hex70b*, *hex100*, *ilp1*, *ilp2*, *vitellogenin*) in both larvae and pupae of *Apis mellifera*. Azoxystrobin hampered the expression of *hex70b*, *hex100*, *ilp1*, *ilp2,* and *vitellogenin* in both larvae (one-way ANOVA, *hex70b*, F(4,10) = 10.23, *p* = 0.0015; *hex100*, F(4,10) =11.97, *p* = 0.0008; *ilp1*, F(4,10) = 6.85, *p* = 0.0064; *ilp2*, F(4,10) = 18.30, *p* = 0.0001; and *vitellogenin*, F(4,10) = 21.06, *p* < 0.0001) and pupae (one-way ANOVA, *hex70b*, F(4,10) = 4.74, *p* = 0.0210; *hex100*, F(4,10) = 25.97, *p* < 0.0001; *ilp1,* F(4,10) = 8.91, *p* = 0.0025; *ilp2*, F(4,10) = 43.47, *p* < 0.0001; and *vitellogenin* F(4,10) = 34.50, *p* < 0.0001). The data in the figure are mean ± SE (standard error) and the different lowercase letters above bars indicate significant difference among different azoxystrobin exposure treatments (*p* < 0.05).

The expression patterns of five nutrient-related genes were altered in the larvae and pupae in response to azoxystrobin exposure (Figure 3). The azoxystrobin affects the expression of *ilp1* in both larvae (one-way ANOVA, F(4,10) = 6.85, *p* = 0.0064) and pupae (F(4,10) = 8.91, *p* = 0.0025), and the expression of *ilp1* in pupae was significantly increased in the 167 mg/L treatment group (*ilp1* in pupae, for 125 mg/L group, *p* = 0.9105; for 167 mg/L group, *p* = 0.0063; for 250 mg/L group, *p* > 0.9999; for 313 mg/L group, *p* = 0.9742), but a further increase in the exposure concentration resulted in a decrease in the expression of *ilp1*. Moreover, the genes *ilp2* (for larva, F(4,10) = 18.30, *p* = 0.0001; for pupa, F(4,10) = 43.47, *p* < 0.0001), *hex100* (for larva, F(4,10) = 11.97, *p* = 0.0008; for pupa, F(4,10) = 25.97, *p* < 0.0001) and *vg* (for larva, F(4,10) = 21.06, *p* < 0.0001; for pupa, F(4,10) = 34.50, *p* < 0.0001) exhibited a similar expression pattern, with increased expression levels in both larvae and pupae after azoxystrobin exposure. Increased expression of *hex70b* in larvae was observed in all treatment groups, particularly upon exposure to the 167 mg/L of azoxystrobin (F(4,10) = 10.23, *p* = 0.0015; for 125 mg/L group, *p* = 0.4401; for 167 mg/L group, *p* = 0.0012; for 250 mg/L group, *p* = 0.3042; for 313 mg/L group, *p* = 0.9500). On the contrary, a decrease in the expression of *hex70b* was detected in pupae with increasing exposure concentration, particularly in the treatment group exposed to 313 mg/L of the fungicide (F(4,10) = 4.74, *p* = 0.0210; for 125 mg/L group, *p* = 0.9911; for 167 mg/L group, *p* = 0.9950; for 250 mg/L group, *p* = 0.1326; for 313 mg/L group, *p* = 0.0427).

### 2.6. Effect of Azoxystrobin Exposure on the ROS Level and Enzyme Activity in Both Larvae and Pupae of Apis mellifera Worker

Increases in the ROS level and SOD activity in larvae were detected in all azoxystrobin treatment groups (one-way ANOVA, for ROS, F(4,10) = 1.58, *p* = 0.2539; for SOD, F(4,10) = 2.54, *p* = 0.1054), but the treated groups did not differ significantly from the control. Also, azoxystrobin exposure affected the ROS level (F(4,10) = 3.70, *p* = 0.0426) and SOD activity (F(4,10) = 0.68, *p* = 0.6222) in pupae, whereas the ROS level in pupae with 313 mg/L treatment group (for 125 mg/L group, *p* = 0.1145; for 167 mg/L group, *p* = 0.3495; for 250 mg/L group, *p* = 0.7034; for 313 mg/L group, *p* = 0.9812) and the SOD activity in pupae with 167 mg/L treatment group (for 125 mg/L group, *p* = 0.9696; for 167 mg/L group, *p* = 0.9456; for 250 mg/L group, *p* = 0.9999; for 313 mg/L group, *p* = 0.9407) was lower than control. The CAT activity in larvae was decreased in all azoxystrobin treatment groups (F(4,10) = 1.02, *p* = 0.4410), whereas the CAT activity in pupae was increased (F(4,10) = 2.74, *p* = 0.0895).

## 3. Material and Methods

### 3.1. Fungicides

According to the statistics obtained from the registration data at China Pesticide Information Network [14], the most widely used commercial formulation in China for controlling pathogens of nectar and pollen plants (rapeseed, alfalfa, rice, and apple) is azoxystrobin SC (250 g/L). Considering the recommended field concentrations and situation, azoxystrobin SC (250 g/L) (Syngenta Crop Protection (Nantong) Co., Ltd. Nantong, China) was employed for evaluating the toxic effects of the fungicide on the larvae and pupae of honeybees. As per the instructions for use, the filed-recommended concentration of azoxystrobin corresponded to 800–2000-fold dilution of the commercial formulation. Therefore, four filed-recommended concentrations of azoxystrobin (125, 167, 250, and 313 mg/L) were selected and diluted with the artificial diet of the larvae. The contaminated artificial diet was stored at −4 °C and utilized within a week.

### 3.2. Honeybee

A total of six healthy honeybee colonies (*A. mellifera*) were reared in the experimental apiary at the College of Bee Science and Biomedicine, Fujian Agriculture and Forestry University (China). In addition, there was no farmland or orchards within a 3 km radius of the apiary. With standard beekeeping practices, these six colonies were maintained free of symptomatic diseases, parasites, and pesticide application over the course of the experiment. The experimental larvae and pupae were obtained as follows: six healthy egg-laying queens from the respective colonies were restricted to a defined area on the corresponding comb with empty cells and then placed in a queen egg-laying controller to lay eggs. After 8 h of egg-laying, these combs with newly-laid eggs were shifted to a separate place within the same colony in a queen excluder. After three days, the 1-day-old larvae from the combs were carefully transferred to the culture plates (96-well tissue, Sangon Biotech (Shanghai) Co., Ltd., Shanghai, China) in the laboratory and reared in a dark incubator (Yiheng Scientific Instruments Co., Ltd., Shanghai, China) at 34 °C ± 1 °C, 95 ± 2% relative humidity (RH).

### 3.3. Experimental Design

According to the standard in vitro indoor feeding method, the healthy 1-day-old larvae were transferred to a plate at a suitable temperature and humidity (34° C ± 1 °C, 95% ± 2% RH), and subsequently divided into a control and four azoxystrobin treatment groups (125, 167, 250, and 313 mg/L). Three replicates were employed per treatment group, and each replicate included 48 larvae. The larvae in the fungicide treatment groups were fed a contaminated artificial diet containing different concentrations of azoxystrobin, while the control group (CK) was fed a normal diet. The larvae were fed three different artificial diets (A, B, and C) at different ages, and the formula of artificial diet was referred to the Chinese agricultural standard: the chemical pesticide guideline on honeybee (*A. mellifera* L.) larval toxicity test (NY/T 3085-2017) [24]—the detailed information is shown in Table S1 (Supporting Information). To elaborate, diet A containing fungicide (20 μL) was used to feed 1-day-old larvae, diet B containing fungicide (20 μL) was also used to feed 3-day-old larvae, and also diet C containing fungicide was used to feed 4-, 5-, and 6-day-old larvae in amounts of 30, 40, and 50 μL, respectively. The fresh artificial diet containing azoxystrobin was changed daily. From the first day of treatment, the numbers of dead larva and pupa was recorded daily and then removed. Meanwhile, the numbers of live pupa and newly-emerged bees were recorded, and the methods for calculating survival, pupation, and emergence rates of honeybees are detailed below:

$$\mathrm{Survival}\;\mathrm{rate}=\frac{\mathrm{Number}\;\mathrm{of}\;\mathrm{survived}\;\mathrm{larva}}{\mathrm{Total}\;\mathrm{number}\;\mathrm{of}\;\mathrm{treat}\;\mathrm{larva}}$$

$$\mathrm{Pupation}\;\mathrm{rate}=\frac{\mathrm{Number}\;\mathrm{of}\;\mathrm{pupae}}{\mathrm{Total}\;\mathrm{number}\;\mathrm{of}\;\mathrm{treated}\;\mathrm{larva}}$$

$$\mathrm{Emergence}\;\mathrm{rate}=\frac{\mathrm{Number}\;\mathrm{of}\;\mathrm{newly}\;\mathrm{emergence}\;\mathrm{adult}\;\mathrm{bee}s}{\mathrm{Total}\;\mathrm{number}\;\mathrm{of}\;\mathrm{pupae}}$$

From each treatment group, fifteen white-eyed pupae and fifteen newly-emerged adult bees (within 2 h after emergence) were randomly sampled, and their average weight was calculated by weighing each individual bee. Moreover, five live larvae or pupae were randomly selected, and then separately pooled to generate a single sample for each replication. Three such pooled samples collected from each concentration treatment group were employed for the subsequent detection of ROS level, enzyme activities, and gene expression.

### 3.4. RNA Isolation and cDNA Synthesis

Total RNA from each sample was extracted using UNlQ-10 Column Trizol Total RNA Isolation Kit (B511321, Sangon Biotech (Shanghai) Co., Ltd., Shanghai, China) as per the manufacturer’s instructions. The sample was rapidly mixed with Trizol (1 mL) at 4 °C, homogenized (60 Hz, 5 min), then centrifuged at 12,000 rpm for 5 min, and the supernatant was transferred to a fresh microcentrifuge tube. Chloroform (0.2 mL) was added to the supernatant, then vortexed for 30 s; after incubation for 3 min, the sample was centrifuged at 12,000 rpm for 15 min at 4 °C. Half the volume of ethanol (100%) was added to the aqueous phase, mixed thoroughly and incubated for 10 min. The mixture was transferred to the adsorption column, then RPE solution (500 μL) was added, centrifuged at 10,000 rpm for 30 s, and the centrifuged liquid discarded. The adsorption column was moved to a fresh micro-centrifuge tube, DEPC-treated ddH_2_O (30 µL) was added and centrifuged at 12,000 rpm for 2 min. The quality and concentration of isolated RNA samples were assessed using NanoDrop One (Thermo Fisher Scientific Inc., Waltham, MA, USA), and then the qualified RNA samples were used for cDNA synthesis using Mighty Script Plus First Strand cDNA Synthesis Master Mix (gDNA digester) following the manufacturer’s protocol (B639252, Sangon Biotech (Shanghai) Co., Ltd., Shanghai, China) with two steps. First, genomic DNA removal; the reaction mixture (15 μL volume) included RNA (2 μL), 5× gDNA digester mix (3 μL), and RNase-free water (10 μL), incubated at 42 °C, 10 min. Second, cDNA synthesis; the reaction mixture (20 μL volume) included reaction products of the first step (15 μL), 4× III M-MLV RT Mix (5 μL), and the reaction conditions included 25 °C for 5 min, followed by 55 °C for 15 min, finally at 85 °C for 5 min. The cDNA was stored at −20 °C for analysis of gene expression.

### 3.5. Gene Expression Detection

The relative expression of genes associated with development (*ecr* and *usp*), nutrition (*ilp1*, *ilp2*, *hex70b*, *hex110*, *vg*), and immunity (*abaecin*, *apidaecin*, *defensin1,* and *hymenoptaecin*) were evaluated in both larvae and pupae by ABI QuantStudio six Flex System (Thermo Fisher Scientific, Waltham, MA, and United States) and the *actin* gene was used as the reference gene [25]. All these gene-specific primers were synthesized by the Sangon Biotech (Shanghai) Co., Ltd., (Shanghai, China) and the detailed information is shown in Table S2 (Supporting Information). The Hieff^®^ qPCR SYBR Green PCR Kit (11202ES08, Yeasen Biotechnology (Shanghai) Co., Ltd., Shanghai, China) was used for real-time PCR according to the manufacturer’s guidelines as follows: the qRT-PCR reaction mixture (10 µL volume) included cDNA (1 μL), TB Green Premix Ex Taq II (2×, 5 μL), gene-specific primer (10 μM, 0.4 μL), ROX Reference Dye II (50×, 0.2 μL), and RNase-free water (3 μL). The reaction conditions included denaturation at 95 °C for 30 s, followed by amplification for 40 cycles of denaturation at 95 °C for 5 s, annealing at 60 °C for 30 s. The melting curve step was performed with the temperature varied from 60 to 95 °C for 10 s at 1 °C increment to ensure consistency and specificity of the amplified product. At least three technical and biological replicates were analyzed for each sample.

### 3.6. Estimation of ROS Levels and Enzyme Activities

The ROS level was determined using an ELISA kit (Yuanju Biotechnology Center, Shanghai, China) and the enzyme activity of SOD (hydroxylamine method) and CAT (ammonium molybdate method) was estimated using commercial kits (Nanjing Jiancheng Bioengineering Institute, Nanjing, China). The experiments were strictly carried out as per the manufacturers’ instructions and the absorbance under different wavelengths (ROS 450 nm; SOD 560 nm; CAT 405 nm) was detected by Thermo Scientific Varioskan™ LUX (Thermo Fisher Scientific Inc., Waltham, MA, USA). For the detection of ROS level, the supernatant (10 μL) was mixed with diluent (40 μL), and then transferred to enzyme label plate from the kit. The standard substances of different concentrations (50 μL) were also transferred to the enzyme label plate. Horseradish peroxidase (100 μL) was added to each well of the plate and incubated at 37 °C for 60 min, after which the reaction solutions were discarded. Wash buffer (400 μL) was added to each well for 1 min, then the wash buffer was discarded. Substrate A (50 μL) and B (50 μL) from the kit were added to each well, shade incubated for 10 min, then the stopping solution (50 μL) was added to stop the reaction. The absorbance of each well at 450 nm was determined. For the detection of CAT activity, the supernatant (100 μL) was added to the culture plates (96-well), and then mixed with regent (2.2 mL) from the kit. The absorbance of the mixed solution at 405 nm was determined. For the detection of SOD activity, the supernatant (50 μL) was added to culture plates (96-well), and then mixed with regent (300 μL) from the kit. The mixture was incubated at 37 °C for 40 min, and the chromogenic agent (2 mL) was added. The absorbance of the mixed solution at 550 nm was determined. The assays were performed in triplicate to ensure consistency of results.

### 3.7. Statistical Analysis

The Ct value of the *actin* reference gene was used to normalize the Ct values of the target genes, and the 2^−ΔΔCt^ method was employed to calculate the relative levels of expression of these genes [26]. All data were expressed as the mean ± standard error of the mean (SE). Data analyses and graphical representation were conducted using GraphPad Prism 8.0 (GraphPad Software, Inc., San Diego, CA, USA). The statistical differences in the rate of survival, pupation, and emergence, as well as body weight, enzyme activity, and gene expression between different fungicide treatment groups and the control group were evaluated by one-way analysis of variance (ANOVA), the post hoc analyses were performed using Tukey-HSD, and the significance level was set at a value of *p* < 0.05.

## 4. Discussion

Azoxystrobin has been widely applied for the control of plant fungal diseases for nearly 30 years [25], and its residues and metabolites have been commonly detected in soil, water, as well as in plants and animals, underscoring the potentially high risks to the environment and organisms [11,15,16]. As an important indicator of environmental conditions and a key pollinator, honeybees inevitably contact this fungicide, while the foraging of contaminated pollen and nectar and their consumption by larvae leads to acute or chronic toxicity to individuals and colonies [4,5,6]. Our results demonstrated that field-recommended concentrations of azoxystrobin reduced the developmental indices of both larvae and pupae, and altered the expression of genes related to development, immunity, and nutrition. These changes would affect the survival and ability of the honey bee to respond to climate change, habitat loss, food sources, parasites, pathogens etc.

Long-term exposure to azoxystrobin reduces the rates of survival and pupation of larvae, particularly at the high exposure concentrations (250 and 313 mg/L), indicating that azoxystrobin exposure negatively affects larval survival, and leads to unsuccessful metamorphosis into pupae (Table 1). Furthermore, azoxystrobin could impede the growth and metabolism processes of pupae and newly-emerged adult bees, resulting in a significant reduction in body weight of both stages (Table 2). The body weight of honeybees is a meaningful predictor of pesticide susceptibility [27], and the decline in the body weights of pupae and newly-emerged adult bees is expected to be impacted by their sensitivity to azoxystrobin [28]. Meanwhile, the survival rate and body weight of larvae were reduced with the increase of azoxystrobin. In addition, a similar phenomenon was observed in the investigation of the effects of Pristine on the survival and development of honeybees in that the survival rate and body weight of larvae were significantly reduced with Pristine exposure [5]. One of the active ingredients in Pristine is pyraclostrobin, a strobilurin fungicide like azoxystrobin. It can hamper the mitochondrial respiration of honeybees, leading to a decrease in ATP production during insect development [29,30]. This requires honeybees to consume more nutrients to overcome this tricky problem, leading to abnormal survival and weight of the larvae and newly emerging adults [2]. These findings are indicative of the chronic toxicity of strobilurin fungicides on the growth and development of honeybee larvae, pupae, and newly-emerged adult bees.

Ecdysteroids play an important role in coordinating various physiological aspects during the larval–pupal and pupal–adult transitions in honeybees [31]. At the onset of metamorphosis in insects, the ecdysone hormone 20-hydroxyecdysone (20-E) binds to the EcR/Usp heterodimer to activate its downstream transcription factors and regulate the physiological and metabolic changes associated with molting [19]; both *ecr* and *usp* are key genes for the transduction of ecdysone signals in insects [19,20]. The results obtained herein demonstrated that azoxystrobin disrupts the expression of *ecr* and *usp* in both larvae and pupae of worker bees, which parallels the negative effect of pyrazoxystrobin, another strobilurin fungicide, on the expression of *ecr* and *usp* (Figure 1) [2]. Azoxystrobin induces the expression of ecr in both larvae and pupae, especially at a concentration of 167 mg/L, indicating that this may be the optimal concentration corresponding to the maximal transcriptional activation and expression of ecr in the larvae and pupae. The induced effect of azoxystrobin on the expression of ecr in high concentration treatment groups (250 and 313 mg/L) was attenuated, which was associated with the negative regulation of high concentration of azoxystrobin on the synthesis and regulatory pathways of *ecr* [32]. Up-regulated expression of *ecr* in both larvae and pupae (Figure 1) implies the growth and development will be faster and of shorter developmental duration. However, this is very different from the results of biological observation due to the ultraspiracle (Usp) protein partners with ecdysone receptor (EcR) in the formation of the ecdysone receptor complex [19] and the developmental delay in *usp* knocked-down bees [31]. Therefore, the decreased expression of *usp* in the larvae and pupae would influence the synthesis of ecdysone receptor complex, leading to developmental delays of larvae and increased mortality rate, even though there is high expression of *ecr* (Table 2) [2,3]. This effect is attributable to the fact that 20-E regulates glycol metabolism to increase glucose levels in the hemolymph via the EcR/Usp heterodimer during metamorphosis [19]. Similarly, 20-E also attenuates the expression of *fushi tarazu* transcription factor 1 (*ftz-f1*), juvenile hormone esterase and other developmental regulatory genes [31]. In the present study, the expression profile of *usp* in larvae and pupae with azoxystrobin exposure was different, which may be related to the metamorphosis of the honeybee from larva to pupa. Generally, the expression of *usp* was low in the larval stage, but it was up-regulated in the pupal stage which promotes pupation and molting [33]. Exposure to azoxystrobin altered the expression profile of *usp* in larvae and pupae, with the expression level of *usp* being lower in larvae and higher in pupae, causing abnormal development of larvae and pupae. Despite the increased expression of *ecr* and *usp* in the pupal stage with the 250 and 313 mg/L treatment groups, exposure would accelerate the transition from the pupal to the adult stage and shorten the developmental period, resulting in the incomplete development of tissues and organs, and also a significant reduction in the body weights of newly-emerged adult bees (Table 1) [2,5].

Antimicrobial peptides (AMPs) are small molecular peptides produced in the hemolymph of insects in response to external stimuli or infection caused by a pathogen [25]. These AMPs are essential components of the humoral immune system of insects due to their broad-spectrum antimicrobial activity and ability to provide an immediate pathogen-specific response to invasive pathogens [34,35]. Four families of AMPs (*abaecin*, *apidaecin*, *defensins,* and *hymenoptaecin*) coordinate with each other to ward off viral, bacterial, fungal, and protozoon pathogens in honeybees and are regulated by the Toll and Imd/JNK (immune deficiency and Jun N-terminal kinase) pathways, which are two important intracellular signaling pathways in insects [34]. Indeed, these AMPs are also impacted by antibiotics, pesticides, and other abiotic factors [36,37]. The results shown significant upregulation of two AMP genes, *abaecin* and *apidaecin,* in the larvae and pupae following exposure to azoxystrobin (167, 250, and 313 mg/L) (Figure 2). This suggests a role for these two genes in the immune response of the larvae and pupae against azoxystrobin [38], which is consistent with the finding of a previous study on *Apis cerana*, although the increased expression of AMP genes is known to incur a physiological cost [1,13]. Furthermore, varied effects on the expression of these AMPs were observed with different concentrations of azoxystrobin, and such changes in expression of immune-related genes may alter the resistance of the larvae and pupae to various biotic and abiotic stresses [10,37]. The expression of *hymenoptaecin* was down-regulated in the larvae following exposure to 125, 167, and 250 mg/L of azoxystrobin, but up-regulated in the pupae (Figure 2) prompting the hypothesis that azoxystrobin stress is likely to increase the sensitivity of larvae to pathogens. This may be associated with the physiological status of larvae and pupae or the intracellular signaling pathways [2,34], and further investigation is warranted to evaluate this hypothesis.

Hexamerin is a key functional storage protein that plays an important role in the growth, development, and reproduction of insects, particularly during larval–pupal metamorphosis [39]. The hexamerins are synthesized and secreted into the hemolymph by its fat body cells at the larval stage [40]. During the process of metamorphosis, these proteins are absorbed by the fat body cells and used to reconstruct the tissues and structures of the pupa [20,21]. The gene *hex70b* is vital for the pupal development that responds to the regulation of juvenile hormones and compensates for insufficient dietary proteins [20,41]. The expression of *hex110* peaks in the larval stage and declines subsequently in the pupal stage; this is because the hexamerin molecules stored in the hemolymph are absorbed by the fat body cells via receptor-mediated endocytosis, implying that this gene acts as a nutritional regulator [21,42]. The expression of hex70b in pupae is negatively correlated with the concentration of azoxystrobin treatment and exhibits a certain dose dependent effect. This would result in nutrient deprivation, malnutrition, and delayed development in the pupal stage. The up-regulation of this gene in larvae, particularly upon exposure to 167 mg/L of azoxystrobin as also that of *hex110,* is suggestive of an enhanced nutrient metabolism and hormone regulation in the larvae as an in vivo response to azoxystrobin stress. In honeybee, the *hex70b* and *hex110* were highly transcribed and expressed in the larval fat body [43]. The up-regulated expression of *hex70b* and *hex110* in larvae with azoxystrobin exposure indicate that azoxystrobin would cause energy metabolism disorders and decrease ATP production during insect development [29,30], thereby requiring them to consume more nutrients such as storage proteins to meet their nutritional and energy needs. In fact, azoxystrobin could modify the physiological processes of the bee fat body [44]. The low concentrations of azoxystrobin may stimulate the fat body to accelerate the decomposition and metabolism of stored proteins. If the concentration of azoxystrobin exceeds a certain level such as 250 and 313 mg/L, it may cause damage to the fat body and affect the gene expression of *hex70b* and *hex110*. However, the abnormal upregulation of *hex 110* in pupae indicates that azoxystrobin exposure causes fat body damage, reflecting the effects reported for two other strobilurin fungicides, picoxystrobin [45] and pyraclostrobin [46], which affect the nutrient absorption and storage functions of the fat body for the pupal stage and lead to loss of weight in the pupae.

The insulin/insulin-like signaling pathway (IIS) in honeybees plays a central role in relaying the nutritional status with respect to the growth, development, lifespan, and behavior of adult worker bees [47]. Two insulin-like peptides encoded by *ilp1* and *ilp2* mediate the nutritional signals and regulate energy metabolism in honeybees, which is increasingly recognized as an important regulator of honeybee lifespan [48] and is closely associated with the nutritional status of individual honeybees [23,49]. The *ilp1* is an early ligand for the insulin signaling pathway and is mainly responsible for the direct sensing of nutrition level and is mainly expressed in fat body [50]. The expression of *ilp1* in both larvae and pupae was up-regulated with low azoxystrobin concentrations (l25 and 167 mg/L) (Figure 3), indicating that both larvae and pupae would enhance the nutritional signaling and improve the metabolism of nutrients to compensate for the negative impact of azoxystrobin on the mitochondrial respiration and energy metabolism [47]. Indeed, azoxystrobin could alter the physiological processes of the bee fat body, the function of which would be damaged at a certain concentration [44]. The decreased expression of *ilp1* in both larvae and pupae was observed in high azoxystrobin concentrations (250 and 313 mg/L), implying that azoxystrobin has a deleterious impact on the fat body at two concentrations. Meanwhile, the low expression level of *ilp1* indicates the inhibition of the signal transduction pathway that senses nutritional status, which in turn leads to abnormal nutrient metabolism and body weight loss (Table 2). Moreover, *ilp2* is a general indicator of nutritional status and complements the function of *ilp1* in nutrient metabolism and energy storage [50]. The up-regulated expression of *ilp2* in both larvae and pupae following exposure to azoxystrobin, with the highest expression of this gene in larvae from the 313 mg/L treatment group (Figure 3), may be related to the decrease of ilp1 expression under this condition. Because *ilp1* and *ilp2* demonstrate complementary functions in nutrient metabolism and energy storage, the increased expression of *ilp2* may to a certain extent alleviate the problem of nutrient metabolism disorders and imbalance caused by a decrease in *ilp1* expression [47,50]. This phenomenon indicates that honeybees cope with the azoxystrobin stress by coordinating different metabolic pathways related to nutrient utilization for ensuring normal growth and development; the utilization of additional stored nutrients toward this end leads to malnutrition and weight loss [3]. However, the expression of *ilp2* in the larvae and pupae exhibited a gradual increase and decrease, respectively, with the increase in exposure concentration; this is likely to be related to the difference in the synthesis and metabolism of nutrients during the metamorphosis of honeybees from the larval to the pupal stages [2,23,48].

**Figure 4:**
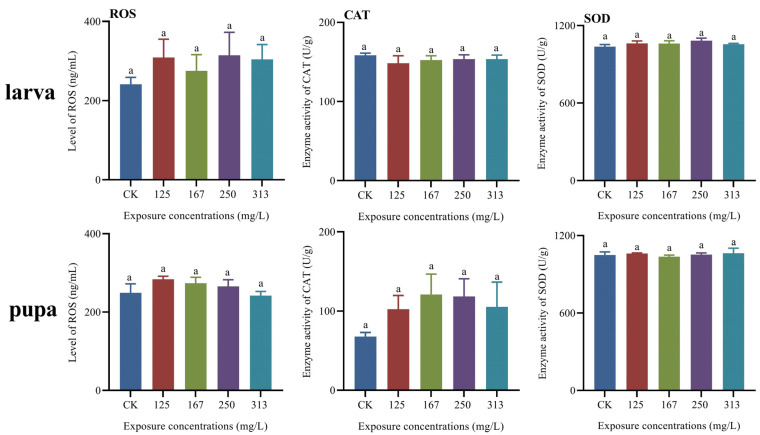
Effect of azoxystrobin on the ROS level and enzyme activity in both larvae and pupae of *Apis mellifera*. Azoxystrobin affected the ROS level and enzyme activity of CAT and SOD in both larvae (one-way ANOVA, ROS, F(4,10) = 1.58, *p* = 0.2539; CAT, F(4,10) = 1.02, *p* = 0.4410 and SOD, F(4,10) = 2.54, *p* = 0.1054) and pupae (one-way ANOVA, ROS, F(4,10) = 3.70, *p* = 0.0426; CAT, F(4,10) = 2.74, *p* = 0.0895 and SOD, F(4,10) = 0.68, *p* = 0.6222).The data in the figure are mean ± SE (standard error) and the different lowercase letters above bars indicate significant difference among different azoxystrobin exposure treatments (*p* < 0.05).

A vast majority of organic pesticides containing cyclic compounds cause changes in reactive oxygen species (ROS) levels in organisms [51]. The primary antioxidant defense enzyme superoxide dismutase (SOD) and catalase (CAT) rapidly convert the excess superoxide anion radicals into water and oxygen [3,15,16]. Accordingly, azoxystrobin containing cyclic structures was found to alter the levels of ROS in zebrafish and earthworms [15,16]. In this study, the ROS level and, the activities of SOD and CAT in the larvae and pupae were also altered with azoxystrobin exposure, and no statistically significant differences were observed (Figure 4). This phenomenon was also observed in the effect of azoxystrobin on male zebrafish in that there were no significant difference in the activities of SOD and CAT in the low concentration treatment groups [15]. There are two possible reasons for this phenomenon: the first reason is that the relatively low concentration of azoxystrobin has limited effects on ROS levels and SOD and CAT activities; the second reason is that the long-term fungicide stress leads to the establishment and utilization of the antioxidant system in developing larvae and pupae to resist the oxidative stress caused by azoxystrobin and to maintain low levels of ROS in their bodies by regulating the activities of the protective enzymes SOD and CAT as well as antioxidants such as vitellogenin (vg) [23,52]. Vitellogenin (Vg) is a protein that provides energy and functionality for insect embryos [3,53]; it is also abundant in honeybee workers and plays a role in various biological processes, including immunity, stress resistance, behavior, and longevity [52,53]. As an antioxidant, vitellogenin prevents senescence and infection in honeybee queens and workers [23]. In the current study, increased expression of *vg* was observed in both larvae and pupae upon azoxystrobin stress, particularly upon exposure to 313 mg/L of the fungicide (Figure 3); this is attributable to the induction of oxidative stress and increased ROS levels in organisms upon exposure to azoxystrobin [15,16,54]. The low level of ROS in both larval and pupal stages suggests that Vg acts synergistically with SOD and CAT to reduce the oxidative stress response caused by azoxystrobin exposure, aiding the defense of the larvae and pupae against ROS over a longer lifespan [2]. Furthermore, an appropriate level of ROS in insects facilitates the regulation of signaling pathways in insect cells, the maintenance of microbial homeostasis, and the enhancement of immunity in insects [55].

## 5. Conclusions

Overall, our comprehensive study provides vital insights into azoxystrobin exposure by assessing the chronic toxicity effects on both larvae and pupae of *A. mellifera*. Exposure to field-recommended concentrations of azoxystrobin reduced the developmental indices of both larvae and pupae and also altered the expression of genes related to development, immunity, and nutrition, which would affect their survival and ability to respond to the external environment and pathogens. Our results contribute to a better understanding of the toxicity of azoxystrobin on the health of the honeybee and provide new insights into the rational application of fungicides during pollination. In addition, it is necessary to re-evaluate the safety and potential risks of the fungicides to bumblebees, solitary bees, and other insect pollinators in the future, which will provide crucial information for the design of sustainable guidelines for pesticide use in agro-ecosystems.

## Figures and Tables

**Table 1:** Effect of azoxystrobin exposure on the rates of survival, pupation, and emergence of larvae and pupae.

Exposure Concentration (mg/L)	Survival Rate (%)	Pupation Rate (%)	Emergence Rate (%)
313	67.83 ± 1.34 ^d^	48.15 ± 2.89 ^c^	89.96 ± 1.74 ^a^
250	82.31 ± 3.62 ^c^	56.72 ± 4.37 ^c^	89.87 ± 1.91 ^a^
167	87.36 ± 0.96 ^b^	67.55 ± 3.60 ^b^	89.36 ± 4.41 ^a^
125	95.53 ± 1.61 ^a^	76.87 ± 2.64 ^a^	92.69 ± 5.69 ^a^
CK	95.71 ± 2.83 ^a^	81.11 ± 5.87 ^a^	93.89 ± 2.99 ^a^

The data in the table are mean ± SE (standard error) and the different lowercase letters in table indicate significant difference among different azoxystrobin exposure treatments (*p* < 0.05).

**Table 2:** Effect of azoxystrobin exposure on the body weight of pupae and newly-emerged adult bees.

Exposure Concentration (mg/L)	Pupa Weight (mg)	Newly-Emerged Adult Bee Weight (mg)
313	133.95 ± 2.78 ^d^	99.60 ± 3.42 ^d^
250	149.80 ± 5.41 ^c^	108.45 ± 1.15 ^d^
167	167.30 ± 7.27 ^b^	119.58 ± 8.12 ^c^
125	168.25 ± 0.82 ^b^	132.95 ± 4.65 ^b^
CK	182.18 ± 5.19 ^a^	154.50 ± 5.16 ^a^

The data in the table are mean ± SE (standard error) and the different lowercase letters in table indicate significant difference among different azoxystrobin exposure treatments (*p* < 0.05).

## Data Availability

The data that support the findings of this study are available from the corresponding author upon reasonable request.

## Supplementary Materials

The following supporting information can be downloaded at: www.mdpi.com/xxx/s1/.
