# Supplementary material for: Azoxystrobin Exposure Impacts on Development Status and Physiological Responses of Worker Bees (Apis mellifera L.) from Larval to Pupal Stages
Source: Int J Mol Sci. 2024 Nov 3;25(21):11806. doi: 10.3390/ijms252111806 (PMC11546862; doi:10.3390/ijms252111806)
Supplement: Supplementary file 1 [file ijms-25-11806-s001.zip › ijms-3203542-supplementary.pdf]

Supplementary material

Formula of the artificial diet for honeybee larvae of different ages (Table S1);

Primers of development, nutrition and immune genes used for qPCR (Table S2).

Formula of the artificial diet for honey bee larvae of different ages

The formula for the artificial diet was according the chemical pesticide guideline on honeybee (*Apis mellifera* L.) larval toxicity test (NY/T 3085-2017) published by the Ministry of Agriculture of the People's Republic of China. The artificial diet is composed of fresh royal jelly, glucose, fructose, yeast extract, and sterile water. For larvae of different age, three different diet formulas (A, B and C) were utilized. The specific formula of the artificial diet was as follows (all in weight ratio):

Table S1. Formula of artificial diet for larvae of different ages

Artificial diet	Larval stage (day-old)	Composition and ratio of artificial diet				
		Royal jelly	Glucose	Fructose	Yeast extract	Water
A	1, 2	50%	6%	6%	1%	37%
B	3	50%	7.5%	7.5%	1.5%	33.5%
C	4, 5, 6	50%	9%	9%	2%	30%

Primers of development, nutrition and immune genes used for qPCR

Table S2. Primers of development, nutrition and immune genes used for qPCR

Genes		Primer sequence (5'→3')[a]	Gene ID
Reference gene	*actin*	F: TTGTATGCCAACACTGTCCTTT<br>R: TGGCGCGATGATCTTAATTT	NM_001185145.1
Development-related genes	*ecr*	F: GTTTGCGTTTGGAAAGTCACG<br>R: GGGGGACCTTTTATGCGTGT	XM_016913298.2
	usp	F: GGCACGAGGTAAAAGTGACGA<br>R: TTAGCCAAGTGTTGCCACGG	NM_001011634.2
Immunity-related genes	*abaecin*	F: CAGCATTCGCATACGTACCA<br>R: GACCAGGAAACGTTGGAAAC	NM_001011617.1
	apidaecin	F: TTTTGCCTTAGCAATTCTTGTTG<br>R: GTAGGTCGAGTAGGCGGATCT	NM_001011613.1
	defensin-1	F: TGCGCTGCTAACTGTCTCAG<br>R: AATGGCACTTAACCGAAACG	NM_001011616.2
	hymenoptaecin	F: CTCTTCTGTGCCGTTGCATA<br>R: GCGTCTCCTGTCATTCCATT	NM_001011615.1
Nutrient-related genes	*ilp1*	F: TGGCAAGGTGTCTATCACCG<br>R: ACGTCAGCAGCATATCACCA	XM_026442143.1
	ilp2	F: TTCCAGAAATGGAGATGGATG<br>R: TAGGAGCGCAACTCCTCTGT	NM_001177903.1
	hex110	F: ACAACAAGCAGGACAACAGGA<br>R: ACCAAGTCCGTTAGAAAGACGA	NM_001101023.1
	hex70b	F: CCTTGGACCGAAATACGACGA<br>R: GTGTTGCTTCCGCTTTTCAGG	NM_001011600.1
	vg	F: AGTTCCGACCGACGACGA<br>R: TTCCCTCCCACGGAGTCC	NM_001011578.1

[a]F, forward primer; R, reverse primer
